# Stability, Homogeneity and Carry-Over of Amoxicillin, Doxycycline, Florfenicol and Flubendazole in Medicated Feed and Drinking Water on 24 Pig Farms

**DOI:** 10.3390/antibiotics9090563

**Published:** 2020-08-31

**Authors:** Femke Vandael, Helena Cardoso de Carvalho Ferreira, Mathias Devreese, Jeroen Dewulf, Els Daeseleire, Mia Eeckhout, Siska Croubels

**Affiliations:** 1 Laboratory of Pharmacology and Toxicology, Department of Pharmacology, Toxicology and Biochemistry, Faculty of Veterinary Medicine, Ghent University, 9820 Merelbeke, Belgium; Femke.Vandael@UGent.be (F.V.); Mathias.Devreese@UGent.be (M.D.); 2 Department of Reproduction, Obstetrics and Herd Health, Veterinary Epidemiology Unit, Faculty of Veterinary Medicine, Ghent University, 9820 Merelbeke, Belgium; Helena.Ferreira@UGent.be (H.C.d.C.F.); Jeroen.Dewulf@UGent.be (J.D.); 3 Research Institute for Agriculture, Fisheries and Food (ILVO), Technology and Food Science Unit (T&V), 9090 Melle, Belgium; Els.Daeseleire@ilvo.vlaanderen.be; 4 Department of Food Technology, Food Safety and Health, Faculty of Bioscience Engineering, Ghent University, 9000 Ghent, Belgium; Mia.Eeckhout@UGent.be

**Keywords:** pig production, oral group medication, medicated feed, medicated drinking water, residues, veterinary drugs, stability, homogeneity

## Abstract

The vast majority of medicines in pig rearing are administered via oral group medication through medicated feed and drinking water. However, relevant on-farm factors affecting the concentration of these drugs in feed and drinking water, such as the homogeneity, stability, and cross-contamination, are largely unknown. To characterize these factors, samples of medicated feed and drinking water were taken on 24 Belgian pig farms during treatment and 2 days thereafter, as well as at different on-farm sampling sites from production to feeding troughs or drinking nipples. The samples contained amoxicillin, doxycycline, florfenicol, or flubendazole. Additionally, a questionnaire was completed. In contrast to the results of medicated feed, results of medicated water showed a large between-farm variation in antimicrobial drug concentration. The therapeutic concentration range was only met in 2 out of 11 farms using medicated feed, and in 3 out of 13 farms using medicated water. Medicated feed concentrations were often below the therapeutic concentration range mentioned in the Summary of Product Characteristics, while drinking water concentrations were just as often above as they were below the advised target concentration range. Drug residues measured 2 days after the end of therapy with both feed and water medication rarely exceeded 1% of the lowest therapeutic concentration. This study demonstrates that recommendations on good clinical practices for oral group medication in the pig industry are highly needed.

## 1. Introduction

Oral group therapy with antimicrobials and anthelmintics is common practice in livestock farming. Especially in pig production, antimicrobial drugs are primarily administered through the feeding and drinking water system [1,2].

Medicated feed in the pig industry in Belgium is prepared either by (a) the compound feed manufacturer at the feed mill, i.e., for antimicrobials via a mixer at the end of the production line, whereas anthelmintics such as flubendazole can also be mixed at the main production line; (b) the compound feed manufacturer at the moment of delivery at the farm using a fine dosing system mounted on the delivery truck; or (c) the pig farmer on the farm using a dosing system on the feed line, or by topdressing [2,3]. Medicated drinking water is always prepared at the farm, using an electrical or mechanical dosing pump that mixes a pre-solution at a set percentage into the drinking water, or using a drinking water reservoir [2]. In our previous survey conducted on 52 Belgian pig farms [2], medicated drinking water and feed were used on 90.4% and 69.2% of the farms, respectively. In the latter case, feed was purchased from a compound feed manufacturer in 68.2% of the cases, topdressing was used on 25.0% of the farms, and a dosing device on the feed line was used on 6.8% of the farms. Medicated drinking water was prepared using a mechanical dosing pump (51.9%), an electrical dosing pump (30.8%), or a drinking water reservoir (30.8%). The same study also concluded that there is considerable room for improvement for oral group treatment by optimizing cleaning and disinfecting protocols used at the farm, developing appropriate pharmaceutical formulations for drinking water medication, performing regular controls of the drinking water quality, and using separate pipeline circuits.

The homogeneity and stability of antimicrobials provided via the feed and drinking water for curative pharmacological treatment is crucial to meet the main pharmacokinetic/pharmacodynamic (PK/PD) cut-off values for clinical efficacy [4], and consequently for animal health, animal welfare, and prudent use of antimicrobials. Homogeneity, stability, and carry-over of veterinary drugs in medicated feed and drinking water are likely influenced by (a) the different methods used to prepare medicated feed and drinking water [4], and to transport and store medicated feed both at the feed factory and at the farm [5]; (b) the materials coming into contact with the medicated feed and drinking water (e.g., materials from feed/drinking water pipelines) [6,7]; and finally, (c) the dose and duration of treatment [8]. Moreover, the quality and physicochemical properties of the drinking water are important for the solubility, homogeneity, and stability of drugs in medicated drinking water [9]. Drinking water quality starts with the source, and is influenced by the quality of the pipelines and water treatment procedures, for example purification or acidification with organic acids aim to diminish infections by enteropathogenic bacteria [10,11]. Yearly testing of drinking water quality is recommended, but previous studies show this is often not the case [2,12].

Although group treatment should be avoided and reduced as much as possible to limit unnecessary exposure of animals, and thereby limiting antimicrobial resistance selection, recommendations on good practices for group treatment are very much needed. In our previous research, we concluded that there are no data available that describe the homogeneity, stability, and carry-over of medicated feed and medicated drinking water prepared on pig farms [2].

Therefore, in the present study, the on-farm homogeneity, stability, and carry-over of commonly used medicinal products in the pig production sector (amoxicillin (AMO), doxycycline (DOX), florfenicol (FLOR), and flubendazole (FLU)) were examined on 24 farms, when mixed in feed and/or drinking water.

## 2. Results

### 2.1. Medicated Feed

Medicated feed samples, collected on 11 pig farms, contained AMO (*n* = 7), DOX (*n* = 3), or FLOR (*n* = 1). Medicated feed was produced at the feed mill with an end-of-line mixer (*n* = 9), or a veterinary medicinal product was mixed by the farmer using a dosing system on the feed line (*n* = 2, farm number 1 and 2, Table 1). There were no farms in which medicated feed was mixed using a fine dosing system at the delivery truck. Whenever medicated feed was bought from the feed mill, it was stored in a silo at the farm. The distance between the silo and first feeding trough ranged between 2 and 50 m (mean ± SD, 16.6 ± 15.6 m). It was not possible to take samples from the silos, as these were not accessible through a hatch. Feed pipelines were made of polyvinyl chloride or PVC (*n* = 4), galvanized steel (*n* = 4), stainless steel (*n* = 2), or a combination of PVC and stainless steel (*n* = 1). Medicated feed was used for piglets on eight farms, and for fattening pigs on three farms. The oldest group receiving medicated feed was 14 weeks old. The size of the group of pigs receiving medicated feed ranged from 175 to 2000 animals (mean ± SD, 670 ± 490 pigs), and they were either housed in one or two stables (*n* = 4) or in a part of a stable (*n* = 7). This meant that on seven farms, one silo was used to feed a part of a stable, instead of the entire stable. The therapeutic indications listed by the veterinarian were coughing (*n* = 2), *Streptococcus suis* infection (*n* = 2), and atrophic rhinitis (*n* = 1), and medicated feed was also administered as a preventive measure (*n* = 6). According to farmers and veterinarians, all pigs were clinically cured in case a therapeutic treatment was installed. A treatment lasted between 3 and 18 days (mean ± SD, 7.7 ± 3.7 days). The concentrations measured in the medicated feed samples are presented in Table 1

Only on two farms (number 8 and 10) were the concentrations of DOX in feed consistently within the therapeutic range. On two other farms (number 2 and 6), the concentrations of AMO were lower than the therapeutic concentration range for all samples. At 2 days after the end of the treatment, no feed samples on any farm had residues exceeding 1% of the lowest therapeutic concentration, as mentioned in the Summary of Product Characteristics (SPC), except for farm number 7 where the concentrations measured were comparable to the concentrations measured during the middle and last day of treatment.

### 2.2. Medicated Drinking Water

Thirteen farms using medicated drinking water were sampled, either using AMO (*n* = 5), DOX (*n* = 5), FLOR (*n* = 2), or FLU (*n* = 1). The medicinal products were mixed in the drinking water using a mechanical dosing pump (*n* = 7), an electrical dosing pump (*n* = 4), or a drinking water reservoir provided either with (*n* = 1) or without (*n* = 1) a stirrer (shown in Table 2. Well water was the most commonly used source of drinking water (*n* = 8), followed by rain water (*n* = 3), tap water (*n* = 1), surface water (*n* = 1), and depth drainage water (*n* = 1). One farm used two different water sources. Pipelines were most often made of PVC (*n* = 11). Stainless steel (*n* = 1) and a combination of the two (*n* = 1) were also observed. None of the farms had a separate pipeline circuit for medicated and non-medicated water. Of the 13 treatments, 7 were used for piglets, and 6 for fattening pigs, which were at most 24 weeks old. Between 120 and 850 pigs were treated as one group (mean ± SD, 488 ± 208 pigs). Of the 13 treatments, 11 were administered to an entire stable, whilst in two cases a part of the stable (comprising a number of compartments) were treated. The antimicrobial drugs were used for curative treatment on eight farms and as preventive measure on four farms. A clinical cure was achieved on seven out of the eight farms. Deworming (*n* = 1) was used as a preventive measure. Treatment period was between 2 and 7 days long (mean of 5.3 days). The veterinary medicinal products were combined with additives on seven farms, consisting of a flavoring agent on two farms, or citric acid to be used with AMO (*n* = 4), or ascorbic acid for use with FLOR (*n* = 1). Samples of the pre-solution, taken just before a new batch was prepared, were between 4 and 60 h old, with a mean of 16 h. Results of the water quality testing are presented in Table 3. At the 12 pig farms administering antimicrobial drugs, the water quality met the requirements for drinking water quality for livestock. Only on farm number 9 were the chloride and sodium concentrations slightly above the recommended levels [13].

The concentrations of the drugs measured in the medicated drinking water are presented in Table 2.

Considering the AMO results at the level of the individual pig farms, the bucket with the pre-solution on farm number 1 had considerable amounts of powder on the bottom. Indeed, the concentration of AMO in the Cpre_stab sample was below the limit of detection. On farm number 2, the concentration was lower than the therapeutic concentration range, without a known explanation. It is probable that the amount of veterinary medicinal product weighed and mixed was too low. On farm number 3, one sample slightly exceeded the therapeutic range. The medicated water on farms number 4 and 5 reached the therapeutic concentration range, wherein the pre-solution remained stable and there were no residues present at levels of >1% of the lowest therapeutic concentration registered for use in Belgium.

When looking at the five farms using DOX in drinking water, only one of them (farm number 7) reached the therapeutic concentration range; three farms were dosing higher, and the medicated drinking water on farm number 6 had concentrations far below the therapeutic concentration range. Residues above 1% of the lowest therapeutic concentration were present on farm number 9, where medication was added to a drinking water reservoir without a stirrer present.

The medicated drinking water prepared on both farms using FLOR (farm number 11 and 12) also had concentrations far below the therapeutic concentration range. The medicinal products were mixed using either an electrical or a mechanical dosing pump. Remarkably, the concentration of FLU measured on farm number 13 was only about 1% of the therapeutic concentration range.

## 3. Discussion

The use of medicated feed and drinking water, especially when containing antimicrobial drugs, is under debate, as it mainly involves group medication [5]. This leads to increased exposure of animals to antimicrobials and a consequent increase of antimicrobial resistance selection pressure of both pathogenic and commensal bacteria [14]. In particular, medicated feed is under debate for being too user-friendly, and therefore being an “easy fix” instead of focusing on prevention of diseases, biosecurity, and other management practices. Therefore, it has been recommended that antimicrobial use be limited as much as possible by reducing the number of treatments and the number of animals treated, as well as by avoiding prophylactic antimicrobial use [15]. This is essential to preserve the efficacy of antimicrobial drugs [16]. If their use is justified, however, the most appropriate antimicrobial drug should be used, on the basis of pharmacokinetic and pharmacodynamic information and following the best clinical practices for prudent use. However, when oral group treatment is needed, no guidelines for proper administration and use are available.

In our recent survey conducted on 52 pig farms in Belgium [2], 69.2% of the farms used medicated feed and 90.4% used medicated drinking water, with 62% of the farms using both. By controlling the concentration of antimicrobial products in medicated feed and drinking water, as well as the quantity delivered and ingested by the animals, therapeutic plasma concentrations can be reached whilst limiting residue formation and antimicrobial resistance [17]. The homogeneity, stability, and residue formation at the farm level are still largely unknown. However, these factors must be understood to select effective reduction measures. To the authors’ knowledge, this study provides the first insights into the on-farm concentrations of antimicrobials in feed, and of antimicrobials and an anthelmintic drug in drinking water.

In this study, medicated feed was mostly used for piglets, whereas medicated drinking water was used for fattening pigs almost as often as for piglets. The mean number of pigs treated was higher when treating with medicated feed than medicated drinking water (670 vs. 488 pigs), and also the treatment duration was longer when using medicated feed compared to drinking water (mean of 7.7 vs. 5.3 days). Medicated feed was also used more for preventive measures (6 out of 11 treatments) compared to medicated drinking water (4 out of 12 treatments using antimicrobial drugs). These results are suggestive that medicated drinking water may be preferable to medicated feed with regard to the number and duration of exposure of animals to antimicrobials. In general, the water quality met the quality requirements, with the exception of one farm where the chloride and sodium content were slightly too high.

The therapeutic concentration range was only met on 2 out of 11 farms using medicated feed, and on 3 out of 13 farms using medicated drinking water. On the other hand, when using water medication, concentrations exceeding the therapeutic range were also observed. This may be the result of an overuse, hence providing the opportunity to reduce the used amount. In general, the length of the pipelines did not have a clear impact on the homogeneity, as demonstrated by the somewhat fluctuating levels measured at the beginning, middle, and end of the pipelines (Table 1 and Table 2). Moreover, the active substances in medicated feed remained stable throughout the treatment period, evidenced by the levels measured at the start, halfway point, and end of the treatment period (Table 1). Remarkably, the results obtained on one farm using a dosing system on the feed line (farm number 2, Table 1) showed almost non-detectable levels of AMO in the samples collected halfway through the treatment period. This farmer forgot to start the dosing system that day. In general, this demonstrates that there is a large amount of improvement possible for both oral group treatment options. Concerning the carry-over results, one farm using medicated feed (farm number 7, Table 1) showed residual concentrations at 2 days after the end of treatment, far exceeding the maximum limit considered acceptable by the Belgian Feed Association after the production of medicated feed batches (set at 1% of the therapeutic concentration) [18]. The reason was that the silo with medicated feed was not completely emptied, resulting in a treatment longer than the veterinarian and farmer had anticipated. Indeed, Ferran and Roques [17] also mentioned the quantity of medicated feed delivered can exceed what is needed, which may result in overuse. Residues at 2 days after cessation of water medication and higher than 1% of the lowest therapeutic concentration were also seen on one farm (farm number 9, Table 2). Here, a drinking water tank without a stirrer was used, most probably resulting in precipitation of the active compound, which remained present for at least 2 days after the end of treatment. Nonetheless, residues lower than 1% of the therapeutic concentration could still give rise to antimicrobial resistance selection pressure, as described for DOX in an in vitro setting, simulating the intestinal environment [19].

In the present study, it was not our aim to determine animal exposure directly by measuring plasma concentrations. Nevertheless, a theoretical exposure during treatment was calculated, as presented by the theoretical mean daily dose and corresponding Cpss shown in Table 1 and Table 2. When evaluating efficacy of the antimicrobials tested, PK/PD indices are used such as AUC/MIC_90_ and T > MIC_90_ (and C_max_/MIC_90_) with MIC_90_ as the minimum inhibitory concentration at 90%, AUC as the area under the plasma concentration time-curve, and C_max_ as the maximum plasma concentration. However, in the present study, only the theoretical Cpss could be calculated on the basis of the available data. Therefore, the Cpss was used as a surrogate PK parameter. Major pathogens include *S. suis* and respiratory infections caused by *Actinobacillus pleuropneumoniae, Bordetella bronchiseptica*, and *Pasteurella multocida*. As mentioned before, the therapeutic indications listed by the veterinarian were coughing, *S. suis* infection, and atrophic rhinitis. MIC_90_ values are presented in Table 4. Considering AMO, the Cpss and MIC_90_ data confirm that the therapy should be sufficient for treatment of *S. suis*, both with medicated feed and drinking water at all farms studied, but not for *A. pleuropneumoniae, B. bronchiseptica*, and *P. multocida*. Regarding DOX, the proposed treatment should only be sufficient for *B. bronchiseptica* and *P. multocida,* except for farm number 6 that had concentrations of DOX far below the therapeutic concentration range in drinking water. With respect to FLOR, therapy should be sufficient for *A. pleuropneumoniae* and *P. multocida*, but not for the other pathogens. It must however be mentioned that this is only a first rough estimate. In order to more accurately relate plasma concentrations to efficacy, blood samples should be collected on the farms and appropriate PK/PD indices for each antimicrobial drug should be calculated.

It must also be mentioned that when examining the SPCs of medicinal products containing the active compounds included in this study, the reported therapeutic concentrations are often very diverse, as was also reported by Postma et al. [29]. For example, medicinal products containing AMO and registered in Belgium for use in drinking water for pigs range from 7.8 mg/kg bodyweight (BW)/day (Amoxy Active^®^, Dopharma, Raamsdonksveer, The Netherlands), to 20.0 mg/kg BW/day (Moxapulvis 50%^®^, VMD, Arendonk, Belgium). Concerning medicated drinking water systems, the use of a dosing pump could be considered superior to a drinking water reservoir [30,31]. Although dosing pumps are frequently used nowadays, a dosing pump is not suitable for every medicinal product. In this study, Amphen^®^ (FLOR granules, Huvepharma, Antwerp, Belgium) was mixed in the drinking water using an electrical (*n* = 1) and mechanical (*n* = 1) dosing pump. To mix Amphen^®^ in water using a dosing pump, according to the SPC, the pump must be set at 20%, which results in a large volume of pre-solution. If the 20% is not set, this may lead to practical issues with poor solubility and precipitation, which may explain the results shown in Table 2. The remarkable results for FLU are probably related to the poor aqueous solubility of the active substance, considering that commercial formulations are suspensions instead of solutions.

This study demonstrates that good clinical practices for the use of medicated feed and drinking water can be advised. These include the continuous education of veterinarians and farmers, emphasizing the importance of a correct preparation of medicated drinking water by following the dose and guidelines mentioned in the SPC; monitoring drinking water quality at regular intervals; and intervening if necessary, using appropriate solubility enhancers, thoroughly mixing the medicinal product, and timely refreshing the pre-solution or changing the dosing pump in such a way that the pre-solution is fully used in a matter of hours. Furthermore, optimizing the protocols for on-farm preparation of medicated feed and drinking water to be included in the SPCs is required, as well as education and training of veterinarians and farmers, demonstrating the importance of fewer treatments and fewer animals treated.

## 4. Materials and Methods

### 4.1. Sample Collection

Specialized pig veterinarians were contacted by e-mail, followed by a phone call explaining the study objectives. Veterinarians willing to cooperate were asked to contact the principal investigator if a treatment with any of the following medicinal products was prescribed via feed or drinking water medication: AMO, DOX, FLOR, FLU, or sulfadiazine/trimethoprim. The farm was only included with the farmer’s consent. Farms using combination therapy were excluded. During the study period, no farms were found using sulfadiazine/trimethoprim. In total, 24 pig farms were visited to collect samples.

During sample collection, a short questionnaire was used to record data concerning (a) the treatment installed (medicinal product, number of treatment days), (b) the pigs being treated (production category, group size (i.e., per pen, per compartment, or per stable), and clinical symptoms (if present)), (c) the feeding and drinking water system (type of feed/water used, pipeline materials, water purification), and (d) the preparation of the medicated feed or medicated drinking water (purchased, or mixed in using a dosing system on the feed line for feed medication; mixed in by an electrical or mechanical dosing pump, or with a drinking water reservoir for water medication). The use of additives for drinking water medication was also recorded, as well as the distance from the silo to the first feeding trough for medicated feed. The full questionnaire (in Dutch) is available upon request.

Feed samples were collected at the beginning, middle, and end of the feeding line, each time on the first, middle, and last day of the treatment, and 2 days after the end of the treatment. The feed samples were taken from the feed troughs by aluminum scoops that were cleaned with tap water and acetone between each sampling occasion. A sample of 500 g was obtained by quartering, according to EC 152/2009 [32]. For this, four samples of each 500 g were taken from two consecutive feeding troughs and combined in one barrel. The barrel was shaken, its content spread on a plastic sheet, and the sample was divided into four equal parts. Two opposite parts were removed and the other two parts were merged again. This was repeated until a 500 g feed sample remained. Feed samples were labeled, double packed in plastic sample bags, and stored at room temperature in the dark until analysis.

Drinking water samples (each time 500 mL) were collected on 1 day during treatment, at the drinking nipples at the beginning, middle, and end of the water line. This was done 3 h after the preparation of a fresh batch of medicated drinking water, and 2 days after the end of the treatment. On the farms using a drinking water tank, samples of 500 mL were also taken at the upper, middle, and lower third of the tank (mean concentration shown in Table 2). Samples from the middle and lower third were collected using a sampling pump (MiniSampler PE, Bürkle, Bad Bellingen, Germany). In case an electrical or mechanical dosing pump was used, a 500 mL sample was also taken from the bucket containing the 3 h old pre-solution. The stability of the active substance in the tank or pre-solution was also investigated by taking a sample just before it was completely used or before a new batch was prepared, and the time (in hours) was noted. Samples were transported in a cool box with frozen cooling elements and with a 12 V connection for further cooling during transport to the laboratory; 3 h was the travel time from the most distant farms. Each 500 mL sample was stored at ≤−15 °C in labeled plastic bottles until analysis. Samples for monitoring the chemical water quality were collected on all farms administering antimicrobial drugs, and samples were collected from nipples not receiving medicated drinking water in recipients, according to the protocol of Animal Health Care Flanders (DGZ, Torhout, Belgium) [13].

### 4.2. Sample Analysis

#### 4.2.1. Reagents and Standards

The analytical standards of AMO, DOX, FLOR, and FLU and the internal standards (IS) ampicillin (AMP) and demeclocycline hydrochloride were all purchased from Sigma-Aldrich (Bornem, Belgium). Amoxicillin-d_5_ (AMO-d_5_) was bought at Santa Cruz Biotechnology (Dallas, TX, USA); threochloramphenicol-d_5_ and oxfendazole-d_3_ were purchased at Witega Laboratorien (Berlin, Germany). All standards were stored according to the recommendations of the supplier. Acetonitrile (ACN) and methanol (MeOH) were of Ultra high Performance Liquid Chromatography (UPLC) grade and obtained from VWR International (Leuven, Belgium). Water was generated from a Milli-Q SP Reagent water system (Merck Millipore, Darmstadt, Germany). Formic acid (FA), acetic acid (AA), and trichloroacetic acid (TCA) were from Merck. Standard stock solutions (SS) for AMO, AMP, AMO-d_5_, FLOR, and threochloramphenicol-d_5_ were prepared in UPLC-water at 1 mg/mL. SS for DOX and demeclocycline were prepared in UPLC-MeOH at 1 mg/mL, and SS of FLU and oxfendazole-d_3_ were prepared in dimethyl sulfoxide (DMSO), pro analysi, at 1 mg/mL. The SS were stored at ≤−70 °C (AMO, AMP, and AMO-d_5_), at ≤−20 °C (DOX, demeclocycline, FLU, and oxfendazole-d_3_), or at 2–8 °C (FLOR and threochloramphenicol-d_5_). Working solutions (WS) were prepared in UPLC-water (AMO, AMO-d_5_, DOX, and demeclocycline) or in UPLC-MeOH (FLU, oxfendazole-d_3_, FLOR, and threochloramphenicol-d_5_) at 10 µg/mL for AMO, AMO-d_5_, DOX, FLU, and oxfendazole-d_3,_ at 20 µg/mL for AMP and demeclocycline, and at 100 µg/mL for FLOR and threochloramphenicol-d_5_. All WS were stored at 2–8 °C, with the exception of AMO, AMP, and AMO-d_5_, which were stored at ≤−70 °C. All SS and WS, except for those of DOX and demeclocycline, were light-sensitive and were shielded from light and stored in dark Eppendorf cups.

#### 4.2.2. Quantification of AMO, DOX, FLOR, and FLU Using LC–MS/MS and UPLC–UV

Analysis of FLOR and FLU was performed at the Technology and Food Science Unit of the Research Institute for Agriculture, Fisheries and Food (ILVO, Melle, Belgium), while AMO and DOX samples were analysed at the Laboratory of Pharmacology and Toxicology of Ghent University (Merelbeke, Belgium). Liquid chromatography coupled to tandem mass spectrometry (LC–MS/MS) methods were developed for the analysis of AMO, DOX, and FLOR in feed, as described further in this section, and for FLOR and FLU in drinking water. For the analysis of AMO and DOX in drinking water, we used liquid chromatography with ultraviolet detection (UPLC–UV) methods. Analysis of the water samples was performed using methods described in an upcoming paper of Vandael et al. (submitted), investigating the stability of the active substances in different types of drinking water.

During visit and sample collection at the farm, the principal investigator did not have access to the prescription data of the veterinarian (posology and name of the commercial formulation used). Hence, in order to evaluate the results obtained, the therapeutic concentration range of a specific active compound was used. The therapeutic concentration range was defined as the lowest and highest therapeutic concentration mentioned in all SPCs of that active substance available for oral use in pigs in Belgium (medicated feed/drinking water), and taking into account the accuracy criterion of the analytical methods used (i.e., −20% to +10%, applied on the lowest and highest concentration, respectively). For medicated drinking water, the therapeutic concentration ranges are 106–368 mg/L for AMO, 82–199 mg/L for DOX, 134–184 mg/L for FLOR, and 134–340 mg/L for FLU.

For AMO in feed, the therapeutic concentration range is 240–550 mg AMO/kg feed. In order to prepare quality control (QC) samples, a premix was used (Dokamox 80%^®^, Ecuphar, Breda, the Netherlands). Blank feed was spiked with premix at 500 mg/kg feed (QCther), and at a residue concentration level (QCres, 1% of the lowest concentration of the therapeutic concentration range, based on the 1% maximum allowed cross-contamination level mentioned in the Convenant for medicated feed of the Belgian Feed Association (BFA)) [18]. Sample preparation consisted of weighing 15.0 g of feed of each study sample in duplicate in a 125 mL plastic container. Next, 60 µL of SS of AMP IS and 30 mL of water were added. The container was shaken for 30 min and then centrifuged at 5250× *g*. The supernatant was transferred to a second container and 30 mL of water was added again to the pellet, and the container was shaken and centrifuged. Both supernatant phases were then combined and mixed. Finally, 25 µL of supernatant, 25 µL of WS of AMO-d_5_ as a second IS, and 950 µL of water were transferred to an autosampler vial. The LC system consisted of an Acquity autosampler combined with an Acquity binary solvent manager, both from Waters (Milford, MA, USA). Chromatographic separation was achieved using an Acquity UPLC BEH C18 (50 × 2.1 mm i.d., 1.7 µm) column, in combination with an Acquity BEH C18 Vanguard cartridge (5 × 2.1 mm; 1.7 µm), both from Waters. The autosampler tray was kept at 5 °C. Mobile phases used for chromatographic separation consisted of ACN (A) and 0.1% formic acid in water (B). The following gradient elution was used: 0–2.5 min (98% A, 2% B), 2.5–2.51 min (74% A, 26% B), 2.51–4.0 min (98% A, 2% B). Flow rate was set at 0.3 mL/min and the injection volume was 1 μL. A Quattro Ultima tandem mass spectrometer from Waters was used for detection, using an electrospray ionization (ESI) probe operating in the positive ionization mode. Masslynx software v. 4.1 was used to quantitate, on the basis of the following MS/MS transitions (*m/z*): 366.2 > 349.1, 366.2 > 207.8 (AMO), and 372.1 > 355.2 (AMO-d_5_).

The therapeutic concentration range for DOX is between 200 and 550 mg DOX/kg feed. As for AMO analysis, QCther and QCres samples were prepared using Doxycycline 75%^®^ (Kela, Hoogstraten, Belgium) at a concentration of 250 mg DOX/kg and 1% of 250 mg/kg, respectively. Sample preparation consisted of weighing 15.0 g of feed in duplicate in a 50 mL Falcon tube, followed by addition of 30 mL of extraction solvent (MeOH/water, 80/20, *v/v*). Next, the mixture was shaken for 30 min and then centrifuged at 5250× *g*. A portion of the above fraction was transferred to an Eppendorf cup, centrifuged again, and the supernatant was filtered using a polyvinylidene fluoride (PVDF) filter (Sigma-Aldrich, Bornem, Belgium). Finally, 25 µL of supernatant, 25 µL of IS WS of demeclocycline, and 950 µL of water were transferred to an autosampler vial. The LC system consisted of an Acquity autosampler combined with an Acquity binary solvent manager, both from Waters. Chromatographic separation was achieved using an Acquity PLRP-S polymeric column (150 mm × 2.1 mm i.d., 3 µm) in combination with a pre-column of the same type (5 mm × 3.0 mm i.d.), both from Polymer Laboratories (Shropshire, UK). The autosampler tray was set at 5 °C. Mobile phases for chromatographic separation were 0.5% formic acid and 0.5% tetrahydrofuran in water (A) and ACN (B). The gradient elution used was as follows: 0.0 min (90% A, 10% B), linear gradient over 3.0 min (50% A, 50% B), 7.0 min (50% A, 50% B), and linear gradient over 15.0 min (90% A, 10% B). Flow rate was set at 0.3 mL/min and the injection volume was 5 μL. A Quattro Ultima tandem mass spectrometer from Waters was used for detection using an ESI probe operating in the positive ionization mode. Masslynx software v. 4.1 was used to quantitate, on the basis of the following MS/MS transitions (*m/z*): 445.4 > 428.1, 445.4 > 154.0 (DOX), and 465.2 > 448.0 (IS).

The same methodology was used for FLOR in feed. According to the SPC, the therapeutic dose is 200 mg FLOR/kg feed, resulting in a therapeutic concentration range of 160–220 mg FLOR/kg feed. QCther samples (200 mg FLOR/kg) and QCres (1% of 200 mg/kg) samples were prepared just before analysis, using the analytical standard. In duplicate, 15.0 g of sample was added to a 50 mL Falcon tube. Next, IS WS was added, followed by 25 mL of ACN/MeOH (95/5, *v/v*). The tube was vortexed and shaken for 30 min, and was then centrifuged for 15 min at 3000× *g*. The supernatant was transferred to another 50 mL Falcon tube and 25 mL of ACN/MeOH (95/5, *v/v*) was added to the pellet again, which was then shaken for another 30 min, followed by a 15 min centrifugation (3000× *g*). The supernatants were combined and vortexed. To 100 µL of the supernatant, we added 4900 µL of buffer solution (H_2_O/ACN/MeOH 50/25/25, *v/v/v* + 0.05% formic acid). After vortex mixing, 1 mL of the solution was filtered using a 0.22 µm filter. The LC system consisted of an Acquity autosampler combined with an Acquity binary solvent manager, both from Waters. For chromatographic separation, an Acquity BEH C18 column (100 × 2.1 mm; 1.7 µm) and an Acquity BEH C18 Vanguard cartridge (5 × 2.1 mm; 1.7 µm) were used, both from Waters. The autosampler tray was set at 5 °C. Mobile phases were water + 0.05% acetic acid (A), and ACN/MeOH (50/50, *v/v*) + 0.05% acetic acid (B). The gradient elution used was 0.0–6.0 min (100% A, 0% B), 6.0–7.0 (100% A, 0% B), and 7.0–9.1 (0% A, 100% B). Flow rate was set at 0.5 mL/min and the injection volume was 5 μL. A Xevo Triple Quadrupole Mass Spectrometer (TQ-MS) instrument was used for detection, using ESI-positive mode unless mentioned otherwise. Masslynx software v. 4.1 was used to quantitate on the basis of the following MS/MS transitions (*m/z*): FLOR 355.78 > 218.87, 355.78 > 184.87, 355.78 > 118.95 (ESI-); chloramphenicol-d_5_ 325.85 > 156.85 (ESI-), FLU 313.86 > 281.89, 313.86 > 122.92, 313.86 > 94.96; and oxfendazole-d_3_ 318.91 > 158.93.

#### 4.2.3. Method Validation

Methods were validated according to the European Commission guideline no. 2002/657 (EC, 2002) [33] and regulation no. 401/2006 (EC, 2006) [34]. Matrix-matched calibration and QC samples were prepared. The following parameters were tested: linearity, within-day accuracy and precision, between-day accuracy and precision, limit of detection, limit of quantification (LOQ), specificity, and carry-over. Main validation results of the methods for analysis of medicated feed can be found in Table S1, and those for the methods for analysis of medicated drinking water will be published in the upcoming article by Vandael et al. (submitted).

### 4.3. Data Processing and Analysis

Data from the survey were coded numerically to assist analysis and entered into Microsoft Excel, 2010, and recoded into categorical data if appropriate. Descriptive statistics were obtained.

The mean concentration of duplicate measurements of the feed and water samples was calculated, which was evaluated with respect to the therapeutic concentration range for the samples collected during treatment.

The theoretical mean daily dose of each antimicrobial drug during treatment (D, mg/kg BW) was calculated on the basis of the average BW and average daily feed or water intake per farm. The latter was based on information presented in [35].

In order to link the results to the MIC_90_ values of major swine pathogens, the theoretical plasma concentration at steady state (C_pss_, mg/L) was calculated for each farm, using the following equation:

C_pss_ = F × D/CL (1)

where F is oral bioavailability, D is mean daily dose (mg/kg·24 h), and CL is total body clearance (L/kg·h). Data for F and CL are based on [28,36,37,38].

## 5. Conclusions

In conclusion, this research provides first insights in the concentrations of active compounds present in medicated feed and medicated drinking water at the pig farm level. Future research should include a larger sampling campaign with more farms per active compound, and also include more active compounds. Next, animal exposure directly measured by plasma concentrations should be considered, as these can then be linked to suitable PK/PD breakpoints. Future studies should not only investigate the on-farm situation, but should also focus on the design or fine-tuning of risk models, such as the one developed by Filippitzi et al. [3] to estimate the risk factors for cross-contamination of non-medicated feed, in order to estimate the magnitude of the risks for reduced homogeneity and stability of medicated feed and drinking water.

## Figures and Tables

**Table 1:** Mean concentrations of amoxicillin (AMO), doxycycline (DOX), and florfenicol (FLOR) in the medicated feed samples (duplicate measurements), presented as µg/g. The samples were collected at the start of treatment period and were sampled at the beginning (Start_b), middle (Start_m), and end (Start_e) of the feed line, as well as halfway through the treatment period (Half, at the beginning (Half_b), middle (Half_m), and end (Half_e), of the feeding line), on the last day of treatment (End, at the beginning (End_b), middle (End_m), and end (End_e), of the feeding line), and 2 days after the end of the treatment to measure the residues (Res) at the beginning (Res_b), middle (Res_m), and end of the feed line (Res_e). Values shown in green are within the therapeutic concentration range for that active compound (240–550 µg/g AMO; 200–550 µg/g DOX; 160–220 µg/g FLOR). Values in red are below the therapeutic concentration range. Values below the detection limit are presented as ND (not detected). The theoretical mean daily dose of each antimicrobial drug during treatment (mg/kg BW) is presented at the bottom, and is based on the average bodyweight (BW) and average daily feed intake per farm. The corresponding theoretical plasma concentration at steady state (Cpss, mg/L) is also presented.

Farm Number	1	2	3	4	5	6	7	8	9	10	11
Active Compound	AMO	AMO	AMO	AMO	AMO	AMO	AMO	DOX	DOX	DOX	FLOR
Start_b	113.3	51.4	240.9	272.3	138.3	237.3	51.5	219.5	153.7	288.6	191.4
Start_m	133.3	212.1	261.2	271.8	235.1	239.5	52.9	378.3	194.0	259.8	195.0
Start_e	126.0	214.0	242.1	261.6	176.4	223.7	50.5	324.5	265.4	263.4	172.4
Half_b	119.1	1.9	241.2	261.1	251.0	235.3	247.4	294.8	233.0	269.7	201.6
Half_m	114.0	ND	239.7	260.7	225.7	239.6	241.4	327.8	191.9	361.9	189.8
Half_e	268.9	ND	238.0	260.2	256.5	235.3	240.2	299.3	193.7	293.3	158.2
End_b	245.6	119.1	251.9	259.3	242.8	199.2	242.3	301.4	157.5	265.5	182.9
End_m	222.0	137.5	245.3	173.0	253.5	213.2	226.0	379.6	117.1	276.1	194.4
End_e	245.1	149.7	271.2	86.7	231.4	235.6	236.7	316.9	179.9	277.2	191.7
Res_b	ND	ND	0.2	0.3	ND	0.9	239.4	ND	ND	ND	0.9
Res_m	ND	0.2	0.7	ND	ND	1.1	251.8	ND	ND	ND	2.2
Res_e	ND	0.3	ND	ND	ND	1.3	109.2	ND	ND	ND	1.0
Theoretical mean daily dose (mg/kg BW)	6.30	4.52	7.93	7.80	7.15	12.83	9.90	8.19	10.51	10.14	4.83
Theoretical Cpss (mg/L)	0.10	0.074	0.13	0.13	0.12	0.21	0.16	0.42	0.55	0.53	0.65

**Table 2:** Mean concentrations of amoxicillin (AMO), doxycycline (DOX), florfenicol (FLOR), and flubendazole (FLU) in the medicated drinking water samples (duplicate measurements), presented as µg/mL. The samples taken during treatment were collected at 3 h after the start of the treatment and at the beginning (Cther_b), middle (Cther_m), and end (Cther_e) of the drinking water pipeline. Both the pre-solution (in case a dosing pump was used), and the drinking water tank were sampled after 3 h (Cpre_3h/Ctank_3h) and just before the pre-solution/tank was finished or just before a new batch of pre-solution/tank was prepared to determine its stability (Cpre_stab/Ctank_stab). Samples were also taken at 2 days after the end of the treatment to measure the residues at the beginning (Cres_b), middle (Cres_m), and end (Cres_e) of the drinking water pipeline. Values shown in green are within the therapeutic concentration range for that active compound (106–368 µg/mL AMO; 82–199 µg/mL DOX; 134–184 µg/mL FLOR; 134–340 µg/mL FLU). Values in red are below the therapeutic concentration range, while values higher than the therapeutic concentration range are shown in yellow. Values below the detection limit are presented as ND (not detected). The type of mixing the medicinal product in the water is also shown. The theoretical mean daily dose of each antimicrobial drug during treatment (mg/kg BW) is presented at the bottom, and is based on the average bodyweight (BW) and average daily water intake per farm. The corresponding theoretical plasma concentration at steady state (Cpss, mg/L) is also presented.

Farm Number	1	2	3	4	5	6	7	8	9	10	11	12	13
Active Compound	AMO	AMO	AMO	AMO	AMO	DOX	DOX	DOX	DOX	DOX	FLOR	FLOR	FLU
Cther_b	149.9	69.5	210.7	206.2	284.2	3.9	166.9	253.7	189.4	220.3	55.8	103.7	2.6
Cther_m	50.9	52.5	379.6	153.8	357.7	9.0	144.1	247.0	278.4	230.4	58.2	107.1	1.9
Cther_e	ND	62.5	154.1	117.0	343.4	5.8	123.5	193.0	143.7	204.4	53.0	77.8	0.5
Cpre_3h	3492	1882	5896	4064	3454	2418	5453	9304	/	/	7032	1217	523.0
Cpre_stab	ND	2927	4972	4094	3561	147	3388	9331	/	/	NA	8023	685.0
Ctank_3h	/	/	/	/	/	/	/	/	172.4	1009	/	/	/
Ctank_stab	/	/	/	/	/	/	/	/	181.2	ND	/	/	/
Cres_b	ND	ND	ND	ND	ND	0.9	0.8	0.2	143.4	ND	ND	ND	ND
Cres_m	ND	ND	0.4	ND	ND	0.7	0.9	0.2	452.5	ND	ND	ND	ND
Cres_e	ND	0.9	ND	ND	ND	0.9	0.8	0.2	30.5	ND	ND	ND	ND
Type of mixing	MP	EP	MP	MP	MP	EP	MP	MP	tank	tank	MP	EP	EP
Theoretical mean daily dose (mg/kg BW)	12.2	5.3	21.9	16.9	34.2	0.59	15.8	38.5	25.5	19.3	6.6	8.8	/
Theoretical Cpss (mg/L)	0.20	0.086	0.36	0.28	0.56	0.031	0.82	2.00	1.32	1.00	0.88	1.17	/

/: not applicable; NA: no sample available; MP: mechanical dosing pump; EP: electrical dosing pump; tank: drinking water reservoir.

**Table 3:** Chemical water quality on 12 pig farms administering antimicrobial drugs via the drinking water. Bold font is used for divergent results. * The reference values were found on the website of “Diergezondheidszorg Vlaanderen” (Animal Health Care Flanders; DGZ, 2019) [13].

Chemical Parameter	Reference *	1	2	3	4	5	6	7	8	9	10	11	12	Mean	SD	Min.	Max.
Ammonium (mg/L)	≤2	0.51	0.16	0.48	0.21	0.14	0.21	0.15	0.25	0.11	<0.10	0.41	<0.10	0.26	0.14	0.11	0.51
Chlorides (mg/L)	≤250	21.9	75.5	15.4	36.32	66.9	36.32	52.84	54.8	**304.3**	36.8	33.4	124.80	71.61	75.52	15.40	304.30
Manganese (mg/L)	≤1	<0.02	<0.02	<0.02	0.16	0.67	0.16	0.12	<0.02	<0.02	<0.02	0.1	0.10	0.22	0.20	0.10	0.67
Sodium (mg/L)	≤400	104	113	38.9	25.8	50	25.8	34.63	28.1	**446.1**	22.9	26.9	35.8	79.33	114.36	22.90	446.10
Nitrates (mg/L)	≤200	<10	<10	<10	<10	11.83	<10	<10	83.31	<10	21.19	<10	38.8	38.78	27.47	11.83	83.31
Nitrites (mg/L)	≤0.5	<0.10	<0.10	<0.10	<0.10	<0.10	<0.10	<0.10	<0.10	<0.10	<0.10	<0.10	<0.10	<0.10	<0.10	<0.10	<0.10
pH (25 °C)	4–9	8	7.6	7.5	7.5	7.5	7.5	6.0	6.2	8	7.1	8.3	7.2	7.37	0.65	6.00	8.30
Sulfates (mg/L)	≤250	16.4	81.74	5.92	76.2	87.53	76.2	69.5	70.1	192.52	44.7	<5.0	61.4	71.11	45.94	5.92	192.52
Total iron (mg/L)	≤2.5	<0.20	<0.20	1.3	0.8	<0.20	0.8	<0.20	<0.20	<0.20	<0.20	<0.20	<0.20	0.97	0.24	0.80	1.30
Total hardness (°D)	≤20	4.4	17.2	12.5	10.8	18.2	10.8	<3.7	9.7	<3.7	18.8	<3.7	9.2	12.40	4.53	4.40	18.80

**Table 4:** MIC_90_ (minimum inhibitory concentration at 90%) values (mg/L) of major swine pathogens for amoxicillin (AMO), doxycycline (DOX), and florfenicol (FLOR), based on [20,21,22,23,24,25,26,27,28].

Pathogen	AMO	DOX	FLOR
*Streptococcus suis*	0.06	16	2
*Actinobacillus pleuropneumoniae*	32	2.34	0.5
*Bordetella bronchiseptica*	4 *	0.5	4
*Pasteurella multocida*	4	0.52	0.5

*: amoxicillin in combination with clavulanic acid.

## Supplementary Materials

The following are available online at https://www.mdpi.com/2079-6382/9/9/563/s1, Table S1: Validation results for the limit of quantification (LOQ), within-day and between-day precision and accuracy experiments for the LC-MS/MS analysis of amoxicillin (AMO), doxycycline (DOX) and florfenicol (FLOR) in pig feed.
