# Supplementary material for: Stability, Homogeneity and Carry-Over of Amoxicillin, Doxycycline, Florfenicol and Flubendazole in Medicated Feed and Drinking Water on 24 Pig Farms
Source: Antibiotics (Basel). 2020 Aug 31;9(9):563. doi: 10.3390/antibiotics9090563 (PMC7559249; doi:10.3390/antibiotics9090563)
Supplement: Supplementary file 1 [file antibiotics-09-00563-s001.pdf]

Supplementary material

Stability, Homogeneity and Carry-Over of Amoxicillin, Doxycycline, Florfenicol and Flubendazole in Medicated Feed and Drinking Water on 24 Pig Farms

Femke Vandael [1], Helena Cardoso de Carvalho Ferreira [2], Mathias Devreese [1], Jeroen Dewulf [2], Els Daeseleire [3], Mia Eeckhout [4] and Siska Croubels [1,*]

[1] Laboratory of Pharmacology and Toxicology, Department of Pharmacology, Toxicology and Biochemistry, Faculty of Veterinary Medicine, Ghent University, 9820 Merelbeke, Belgium; Femke.Vandael@UGent.be (F.V.); Mathias.Devreese@UGent.be (M.D.)

[2] Department of Reproduction, Obstetrics and Herd Health, Veterinary Epidemiology Unit, Faculty of Veterinary Medicine, Ghent University, 9820 Merelbeke, Belgium; Helena.Ferreira@UGent.be (H.C.d.C.F.); Jeroen.Dewulf@UGent.be (J.D.)

[3] Research Institute for Agriculture, Fisheries and Food (ILVO), Technology and Food Science Unit (T&V), 9090 Melle, Belgium; Els.Daeseleire@ilvo.vlaanderen.be (E.D.)

[4] Department of Food Technology, Food Safety and Health, Faculty of Bioscience Engineering, Ghent University, 9000 Ghent, Belgium; Mia.Eeckhout@UGent.be (M.E.)

* Correspondence: Siska.Croubels@UGent.be (S.C.); Tel.: +32-9-264-73-45

Table S1. Validation results for the limit of quantification (LOQ), within-day and between-day precision and accuracy experiments for the LC-MS/MS analysis of amoxicillin (AMO), doxycycline (DOX) and florfenicol (FLOR) in pig feed.

Within-Day Precision and Accuracy (n = 6)									
Analyte	Concentration low (LOQ)			Concentration medium			Concentration high		
	Theoretical concentration (µg/g)	Precision (RSD, %)	Accuracy (%)	Theoretical concentration (µg/g)	Precision (RSD, %)	Accuracy (%)	Theoretical concentration (µg/g)	Precision (RSD, %)	Accuracy (%)
AMO	2.5	5.7	-14.6	25	9.3	-1.9	250	3.2	1.1
DOX	1.0	3.0	-0.7	100	6.2	4.3	200	4.1	-0.3
FLOR	2.0	10.3	5.0	100	10.2	-0.6	200	3.8	6.6
Between-Day Precision and Accuracy (n = 6)									
AMO	2.5	18.3	-9.6	25	7.5	1.5	250	4.8	-1.9
DOX	1.0	3.8	9.0	100	6.4	-0.2	200	5.0	3.7
FLOR	2.0	5.1	-6.2	100	6.8	9.6	200	10.1	-4.1

Note: Acceptability ranges for accuracy: −20% to +10% for conc. ≥10 ng/g, within-day precision: RSD_{max} = 10% for conc.≥100 ng/g, between-day precision: $RSD_{max} = 2^{(1-0.5\log Conc)}$
